# Supplementary material for: Effectiveness and Safety of Ayurvedic Medicines in Type 2 Diabetes Mellitus Management: A Systematic Review and Meta-Analysis
Source: Front Pharmacol. 2022 Jun 8;13:821810. doi: 10.3389/fphar.2022.821810 (PMC9213670; doi:10.3389/fphar.2022.821810)
Supplement: Supplementary file 1 [file DataSheet1.zip › Supplementary Material/Forest and Funnel Plots/Nigella sativa/LDL-C.pdf]

Nigella sativa - LDL-C

Study	Intervention N	Intervention Mean	Intervention SD	Comparator N	Comparator Mean	Comparator SD	Mean Diff. with 95% CI	Weight (%)
Najmi 2012	40	117.88	20.41	40	128.24	12.58	-10.36 [-17.79, -2.93]	50.44
Hosseini 2013	35	168.60	34.00	35	170.20	35.40	-1.60 [-17.86, 14.66]	10.53
Hadi 2015	23	98.10	30.50	20	108.00	28.07	-9.90 [-27.52, 7.72]	8.97
Heshmati 2015	36	107.20	46.20	36	147.60	50.10	-40.40 [-62.66, -18.14]	5.62
Kooshki 2020	27	104.70	34.20	23	111.10	47.60	-6.40 [-29.14, 16.34]	5.38
Jangjo-Borazjani 2021	20	76.44	21.38	20	88.80	17.43	-12.36 [-24.45, -0.27]	19.05
**Overall**							-11.25 [-16.53, -5.98]	

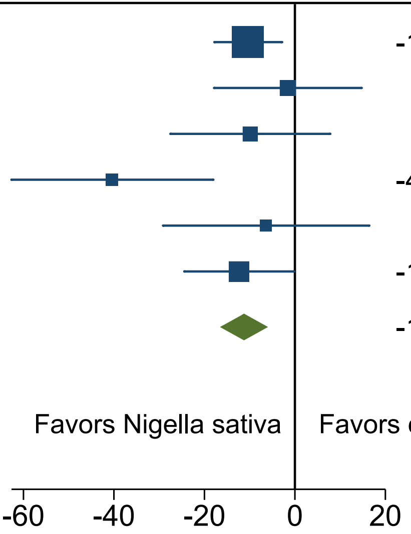

Heterogeneity: τ^2 = 0.00, I^2 = 0.00%, H^2 = 1.00

Test of $\theta_i = \theta_j$: Q(5) = 8.22, p = 0.14

Test of θ = 0: z = -4.18, p = 0.00

Random-effects REML model
